# Latent Profiles and Predictors of Transfer-Assistive Robot Acceptance among Korean Care Workers

**DOI:** 10.1093/geroni/igaf122.4306

**Published:** 2025-12-31

**Authors:** Hee Jeong Yoon, Young Sun Kim

**Affiliations:** Kyung Hee University, Yongin-si, Kyonggi-do, Republic of Korea; Kyung Hee University, kyonggi-do, Kyonggi-do, Republic of Korea

## Abstract

In South Korea, with population aging and a growing shortage of care workers, the adoption of AgeTech in care settings has been steadily increasing, and transfer-assistive robots are increasingly considered to reduce care workers’ workload. This study aimed to identify technology acceptance typologies for transfer-assistive robots among Korean care workers and to examine factors influencing these profiles. In 2023, survey data were collected from 421 care workers engaged in transfer tasks. Latent profile analysis (LPA) across six domains—self-efficacy, anxiety, attitude, ease of use, usefulness, and intention—identified three distinct groups: (a) high-acceptance (proactive) group (53%), (b) anxiety-dominant but moderate-acceptance group (42%), and (c) low-acceptance (prospective) group (5%). Personal characteristics (gender, age, education), health characteristics (depression), job-related burden factors (job burnout, job stress), and technology-related attitude (technology enthusiasm) significantly predicted group membership. The low-acceptance group (c) was more likely to be female, have lower education, higher depression, and lower technology enthusiasm than groups (a) and (b). The anxiety-dominant group (b) was older, had higher job stress, and lower technology enthusiasm compared to the high-acceptance group (a). These findings indicate that technology acceptance patterns among care workers are heterogeneous and that personal, health, job-related burden, and technology-related factors should be considered comprehensively when examining transfer-assistive robot adoption. Understanding these patterns and their predictors can guide tailored implementation and training strategies, supporting the effective adoption of transfer-assistive robots in care settings and informing the development of customized interventions to enhance robot acceptance among older adult care workers.

